# Facile Synthesis of Polymer-Reinforced Silica Aerogel Microspheres as Robust, Hydrophobic and Recyclable Sorbents for Oil Removal from Water

**DOI:** 10.3390/polym15173526

**Published:** 2023-08-24

**Authors:** Zhiyang Zhao, Jian Ren, Wei Liu, Wenqian Yan, Kunmeng Zhu, Yong Kong, Xing Jiang, Xiaodong Shen

**Affiliations:** 1 College of Materials Science and Engineering, Nanjing Tech University, Nanjing 210009, China; 2 Jiangsu Collaborative Innovation Center for Advanced Inorganic Function Composites, Nanjing 210009, China; 3 State Key Laboratory of Materials-Oriented Chemical Engineering, Nanjing 210009, China; 4 Swiss Federal Laboratories for Materials Science and Technology, EMPA, Überlandstrasse 129, 8600 Dübendorf, Switzerland

**Keywords:** silica aerogel, microsphere, polymer crosslinking, hydrophobic, robust strength, oil adsorption

## Abstract

With the rapid development of industry and the acceleration of urbanization, oil pollution has caused serious damage to water, and its treatment has always been a research hotspot. Compared with traditional adsorption materials, aerogel has the advantages of light weight, large adsorption capacity and high selective adsorption, features that render it ideal as a high-performance sorbent for water treatment. The objective of this research was to develop novel hydrophobic polymer-reinforced silica aerogel microspheres (RSAMs) with water glass as the precursor, aminopropyltriethoxysilane as the modifier, and styrene as the crosslinker for oil removal from water. The effects of drying method and polymerization time on the structure and oil adsorption capacity were investigated. The drying method influenced the microstructure and pore structure in a noteworthy manner, and it also significantly depended on the polymerization time. More crosslinking time led to more volume shrinkage, thus resulting in a larger apparent density, lower pore volume, narrower pore size distribution and more compact network. Notably, the hydrophobicity increased with the increase in crosslinking time. After polymerization for 24 h, the RSAMs possessed the highest water contact angle of 126°. Owing to their excellent hydrophobicity, the RSAMs via supercritical CO_2_ drying exhibited significant oil and organic liquid adsorption capabilities ranging from 6.3 to 18.6 g/g, higher than their state-of-the-art counterparts. Moreover, their robust mechanical properties ensured excellent reusability and recyclability, allowing for multiple adsorption–desorption cycles without significant degradation in performance. The novel sorbent preparation method is facile and inspiring, and the resulting RSAMs are exceptional in capacity, efficiency, stability and regenerability.

## 1. Introduction

Oil separation from water is of significant value in saving deteriorating environments. Many research studies have been carried out to develop porous materials for oil adsorption. Among the state-of-the-art porous sorbents, hydrophobic silica aerogels have large specific surface areas, nanopore volumes and porosities, and show excellent performance in adsorption pertaining to water treatment [1,2,3,4]. The methyl groups of the hydrophobic silica aerogels enhance the interaction between silica aerogel and organic contaminants, improve the sorption selectivity and capacity of the organics, and avoid the network failure from water [5,6,7]. Qin et al. synthesized hydrophobic silica aerogel powder by modifying TEOS-derived silica gel with trimethylchlorosilane (TMCS) [8]. The 12 h-modified sample had a specific surface area of 875 m^2^/g, a pore volume of 0.56 cm^3^/g, and a phenol absorption capacity of 142 mg/g from water. Cok et al. investigated the effect of hydrophobic modifier on the structure and organic sorption performance of hydrophobic silica aerogel monoliths [9]. TMCS was functionalized as the surface modifier, and the resulting hydrophobic silica aerogel had a specific surface area and pore volume. The organics and oil absorption capacities were 7.0 and 8.5 g/g, respectively, and were stable for six cycles.

It was found that the expensive organic precursors used in these studies also include some with potential safety and environmental risks, such as ethanol and methanol, which are commonly used as solvents. As an alternative precursor, sodium silicate is low-cost, safe, and environmentally friendly, and has broad raw material resources. Particularly, water is used as the solvent for sodium silicate-based sol–gel systems, which is safer and greener [10]. The sodium silicate-derived silica aerogels with TMCS as hydrophobic modifier achieved a specific surface area of 870 m^2^/g and a pore volume of 4.27 cm^3^/g, which were as high as those of the silicon alkoxide-based counterparts [11,12,13]. The sodium silicate-based hydrophobic silica aerogel monolith showed stable heptane absorption capacity of 3.0 g/g for 10 cycles [14]. The sodium silicate-based hydrophobic silica aerogel powder had a silicone oil absorption capacity of 1.32 g/g [15].

Until now, the hydrophobic silica aerogels have been mostly monoliths, lumps and powders, which are difficult to use in practical projects. And, the aerogel powders used in flow-through separation systems could lead to high backpressure and low permeability [16]. The monoliths are adverse to mass transfer, leading to low sorption capacity owing to a short-circuiting effect. Microspheres, are a new form of aerogel, can avoid the blockage of the fixed adsorbent bed and offer good interaction between the adsorbent and adsorbate [17,18,19,20]. Moreover, the microspheres are easy to recycle from water. However, the innate frangibility of the silica aerogels is still insurmountable, which definitively restricts their recycling. Leventis et al. found that polymer crosslinking could enhanced the strength of silica aerogels [21]. The strength of silica aerogel monoliths has been improved by a factor of over 100 through crosslinking the nanoparticle building blocks of preformed silica hydrogels by using poly (hexamethylene diisocyanate). The resulting polymer coatings can be effectively increased in strength by more than two orders of magnitude. Many polymer agents have already been used as specific chemistries to develop the crosslinking skeletal network; they include polyurea [22], polyurethanes [23], epoxies [24] and polystyrene [25]. However, due to the presence of relatively unstable methyl groups, although their mechanical performance improved, their hydrophobic properties were relatively poor. Among them, polystyrene could exhibit hydrophobicity of 120°, considering that it does not contain atoms or groups that could form hydrogen bonds with water, which is the big difference apart from analogous polyurea and epoxy crosslinked aerogels [26].

Polystyrene, which contains phenyl groups with rigidity and good hydrophobicity, can be used to enhance both mechanical performance and hydrophobic properties. However, the polystyrene reinforced silica aerogel spheres are rarely reported. Besides, there are also few reports about their applications especially in oil removal field. In this work, silica gel microspheres were first prepared from sodium silicate by combining the ball-dropping technique and sol–gel method, which was reported in detail in our previous work [27]. Amino silica gel microspheres were synthesized by grafting amino groups onto the silica gel. Reinforced silica gel microspheres were synthesized after the styrene crosslinking onto the amino gel to form the polymer coatings. The reinforced gel with different crosslinking time was then dried via both vacuum drying (VD) and supercritical CO_2_ drying (SCD) to obtain the reinforced silica aerogel microspheres (RSAMs). Thus, the obtained samples were denoted as R-S-x and R-V-x according to the drying method, where x represents a crosslinking time varying from 3 to 24 h. The effects of different drying methods and crosslinking times on the physical properties, hydrophobicity, chemical structure, pore structure, micromorphology, mechanical performance and oil adsorption performance were investigated.

## 2. Materials and Methods

### 2.1. Materials

Sodium silicate aqueous (33 wt%, modulus 3.3) was supplied by the Que Chen Silicon Chemical Co., Ltd., Wuxi, China. Acetic acid (HAc, 99.5%) was purchased from the Shanghai Shen Bo Chemical Co., Ltd., Shanghai, China. Aminopropyltriethoxysilane (APTES, 98%), simethicone (viscosity 1000 ± 80 mPa·s), vinyl benzyl chloride (90%) and styrene (99.9%) were purchased from the Shanghai Aladdin Biochemical Technology Co., Ltd., Shanghai, China. Azodiisobutyronitrile (AIBN, 99%) and tetrahydrofuran (THF, 99.9%) were obtained from the Shanghai Macklin Biochemical Co., Ltd., Shanghai, China. Anhydrous ethanol was purchased from Wuxi Ya Sheng Chemical Co., Ltd., Wuxi, China. Acetonitrile, n-hexane and vacuum pump oil were purchased from the Sinopharm Chemical Reagent Co., Ltd, Shanghai, China. Vegetable oil (colza oil) was obtained from the domestic daily market. Deionized water was homemade.

### 2.2. Synthesis of the SAMs

**Figure 1:**
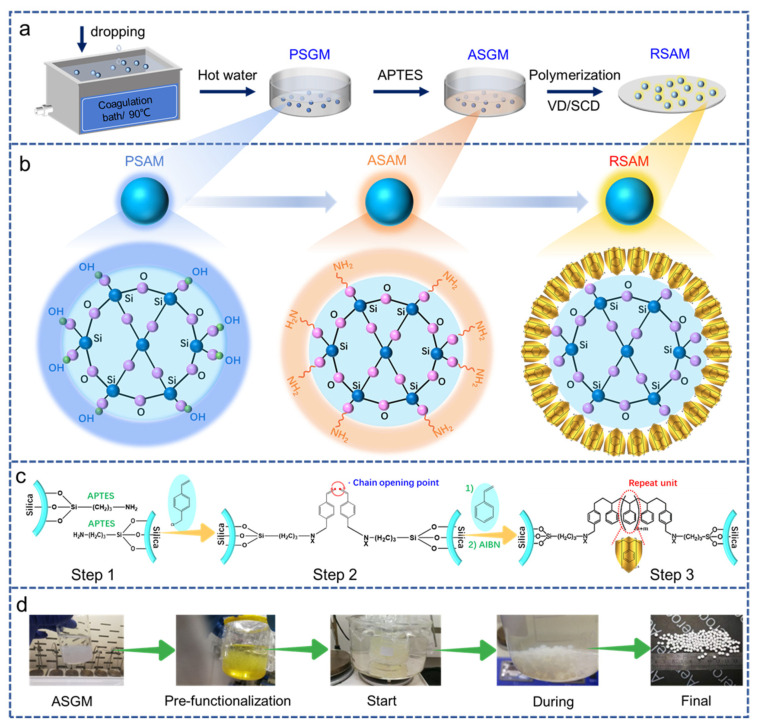
The mechanism of the polymer-reinforced silica aerogel microsphere sol–gel reaction process. (**a**) Facile preparation process. (**b**) Structure of pure, amino, and reinforced aerogels. (**c**) Mechanism of polystyrene crosslinking to form the robust “golden shield”. (**d**) Typical photos of different stages of the polymerization reaction from ASGMs to RSAMs.

Synthesis of the pure silica gel microsphere (PSGMs) and amine-grafted silica gel microsphere (ASGMs). The SGMs were synthesized via the ball-dropping sol–gel method in Figure 1a and reported in detail in our previous work [27]. Typically, an aqueous solution containing 6 mL of sodium silicate, 33 mL of water and 1.2 mL of HAc was prepared at room temperature and then dropped into a silicon oil bath at 90 °C to form the gel within 15 min. Then, the wet gel was fished out from the oil bath and washed with hot water at 90 °C to remove the surface oil. Subsequently, solvent exchange was conducted to replace water inside the wet gel with ethanol. The as-prepared SGMs were immersed in an APTES/H_2_O/EtOH solution with a molar ratio of 1:3:24 for amine grafting at 50 °C for 72 h. Finally, the ASGMs were obtained by flushing with ethanol to remove the residual APTES.

Synthesis of the reinforced silica aerogel microsphere (RSAMs). Briefly, 10 g of ASGM was placed in a container containing 1.3 mL of THF and 8.7 mL of vinyl benzyl chloride and allowed to react at 45 °C for 72 h. The resulting styrene-functionalized SGMs were washed with ethanol three times for 6 h. The styrene-functionalized SGMs were placed in a glass beaker at 50 °C containing 10 mL of styrene, 0.4 g of AIBN and 33 mL of ethanol to perform styrene crosslinking for different time durations varying from 3 to 24 h along with magnetic agitation. The resulting styrene crosslinked SGMs were washed with ethanol three times in 24 h and dried to obtain the RSAMs, as shown in Figure 1d. Both SCD and VD were applied to prepare the RSAMs, and the corresponding samples are denoted as RSAM-S and RSAM-V, respectively. The supercritical CO_2_ drying was performed on a Helix 2 Liter System (Applied Separations, Inc., Allentown, PA, USA) at 50 °C and 100 bar with a CO_2_ gas flow of 0.3 L min^−1^ for 6 h. The vacuum drying was performed at room temperature and −0.1 MPa for 24 h. As a reference, PSAMs and ASAMs were also prepared.

### 2.3. Characterization

The apparent density was calculated from the weight to volume of the SAMs. For each sample, 10 microspheres were selected randomly to measure the average values. Fourier transform infrared (FTIR) spectroscopy was performed on a Shimadzu IR-Spirit Fourier transform infrared spectrometer with an attenuated total reflection (ATR) kit. The thermogravimetric/differential thermal analysis (TGA/DTA) was performed on a Henven HCT-1 thermogravimetric analyzer with air flow of 50 mL/min. The water contact angle was measured on a JC-2000D1 contact angle instrument. Scanning electron microscope (SEM) images were taken on a JEOL JSM-7600F field emission scanning electron microscope. The N_2_ adsorption/desorption test was performed on a Belsorp miniX porosimeter. The specific surface area was calculated by the BET method, while the pore size distribution was obtained by the BJH model from the desorption branch. The compression test was performed on an MTS CMT4204 universal testing machine, the loading cell was 1000 N and the loading speed was maintained at 0.1 mm/min. The oil adsorption capacity was evaluated with pump oil, diesel, gasoline, chloroform, and hexane via a gravimetric method. Sorbents were placed in the oils for 5 min and then isolated to weight. Each test was repeated three times to calculate the mean adsorption capacity. For the regeneration process, oils were desorbed by ethanol washing first and vacuum drying at room temperature.

## 3. Results and Discussion

### 3.1. Synthesis Mechanism

The whole preparation mechanism of the polymer-reinforced silica aerogel microsphere sol–gel reaction process is shown in Figure 1. The detailed preparation process for wet microspheres can be found in our previous work [27]. As shown in Figure 1a, by combining the sol–gel reaction and ball-dropping method, the pure silica gel microspheres (PSGMs) were obtained in the primary step. Subsequently, through APTES modification, the amino-modified SGMs (ASGMs) were obtained. Furthermore, these ASGMs underwent the styrene crosslinking process and were dried via both VD or SCD, resulting in the reinforced silica aerogel microspheres (RSAMs). The structural evolution of different microspheres after drying is shown in Figure 1b. PSAM represents the pure one, the surface of which is covered with hydrophilic hydroxyl groups. ASAM represents these silica microspheres with amino groups grafted onto the surface. The APTES facilitated the substitution of a significant number of hydroxyl groups with amino groups. These amino groups are then pre-functionalized with vinyl benzyl chloride to yield styrene-functionalized wet gels. The AIBN was functioned as an initiator to open the polymer chains, while the styrene monomer acted as an additive to expand the polymer chains. Consequently, a dense polystyrene layer was formed on the surface of the silica microspheres, effectively encapsulating the delicate silica core like the robust golden shield. The mechanism of this golden shield reaction is vividly depicted in Figure 1c. Notably, the dense polystyrene polymer not only enhances the strength but also imparts hydrophobicity. Figure 1d shows the different stages of the polymerization reaction, especially the significant stage of polymerization from ASGM to RSAM. The first picture is of the amino-modified silica gel microspheres (ASGMs), which are surrounded by abundant -NH_2_ groups and rolled as the precursors in the polymerization reaction. The second picture is of the pre-functionalization process; these amino groups on ASGMs are pre-functionalized with vinyl benzyl chloride to yield styrene-functionalized wet gels, a process during which the styrene groups are grafted onto the main body of the silica gel. The third and fourth pictures are of the beginning and duration states of the polymerization reaction of the styrene monomer under the initiator AIBN. Twenty-four hours is the optimal duration to both acquire robustness and maintain sphericity at the same time, and excessive crosslinking time will lead to macroscopic crosslinking of spherical materials, gradually forming a monolithic structure, as shown in Figure S1, resulting in the inability to obtain the single spherical material. The last picture in Figure 1d is of the obtained RSAMs, which exhibit a high degree of sphericity.

### 3.2. Physical Properties

**Figure 2:**
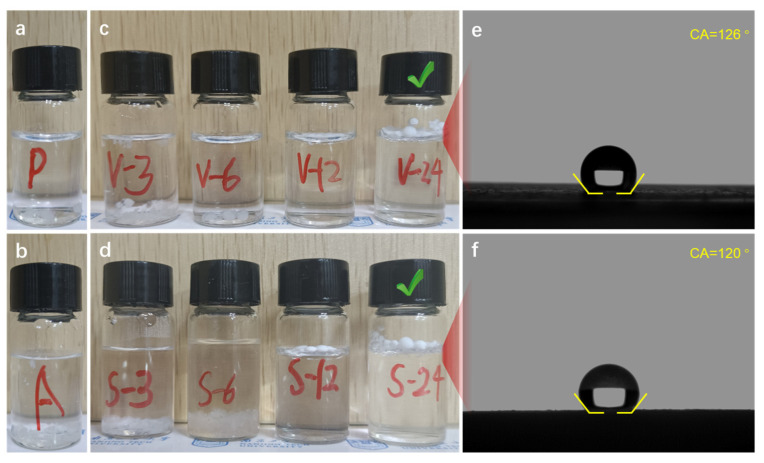
Photographs of aerogel samples in water and the water contact angle (WCA) of hydrophobic samples. (**a**) PSAMs. (**b**) ASAMs. (**c**) RSAMs under different crosslinking times via VD method. (**d**) RSAMs under different crosslinking times via SCD method. (**e**) WCA of R-V-24. (**f**) WCA of R-S-24.

The apparent densities of SAMs are presented in Table 1. All of the SAMs possessed uniform appearance and perfect sphericity, although through different drying methods. The PSAM and ASAM samples showed light weights of 0.080 and 0.137 g/cm^3^, respectively. For the RSAMs, the apparent densities obtained via vacuum drying were much larger than those via supercritical CO_2_ drying. It is understandable when the volume shrinkage of the SGM during the drying process is taken into consideration under different drying methods. Additionally, the apparent density gradually increases with the increasing crosslinking time from 3 to 24 h, revealing more polymer species loaded onto the framework of the aerogel skeleton. Thus, RSAM samples crosslinking for 24 h exhibited the maximum density and the denser appearance, especially the R-V-24 sample, the density (0.21 g/cm^3^) of which was much higher than that of traditional silica and silica-based aerogels (~0.1 g/cm^3^) [28,29,30]. The digital photos of the PSAMs, ASAMs and RSAMs in water, obtained via different drying methods under different crosslinking times, are vividly shown in Figure 2. Both the PSAMs and ASAMs show completely hydrophilic properties in Figure 2a,b, considering that they are surrounded by non-hydrophobic hydroxyl and amino groups, whose structures are explained in Figure 1b. It can be seen that, for SAMs, at the beginning they also exhibited hydrophilic properties and stayed at the bottom in water. With the crosslinking time increased from 6 to 12 h, the samples were able to approach the surface of the water, indicating they were gradually changing from completely hydrophilic to hydrophobic. When the crosslinking time approached 24 h, the RSAMs possessed the highest water contact angle of 126°. The RSAMs synthesized via the VD method exhibited slightly higher water contact angles than those synthesized via the SCD method. However, there were no obvious connections between the water contact angle and synthesis technologies like the drying method. The hydrophobicity of the aerogel is mainly dependent on the surface energy and microstructure [31]. On one hand, the surface energy is closely related to the length of the hydrophobic chain. The longer the hydrophobic chain, the lower the surface energy, resulting in better hydrophobicity of the aerogel. On the other hand, the surface of the micro-nano structure can create a certain micro-roughness, which is beneficial to improve the hydrophobicity. And, compared with the well-known methyl groups, phenyl groups derived from polystyrene have longer hydrophobic chains. Therefore, with the increase in crosslinking time, more and more phenyl groups wrapped onto the aerogel skeleton like a dense shield. Thus, the hydrophobicity of the aerogel gradually increased.

### 3.3. Chemical Structure

**Figure 3:**
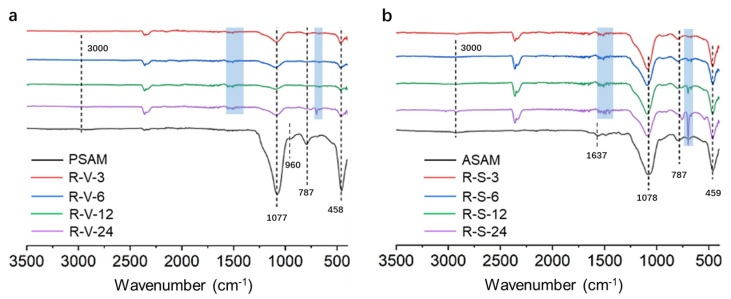
FTIR spectra of the RSAMs obtained by different drying methods. (**a**) VD. (**b**) SCD.

The FTIR spectra of the PSAMs, ASAMs and RSAMs obtained by different drying methods are shown in Figure 3a,b. All of the SAM samples had three bands around 450, 787 and 1077 cm^−1^, which were attributed to the deformation vibration of O-Si-O, symmetric stretching vibration of Si-O (Si-O-Si) and antisymmetric stretching vibration of Si-O-Si, respectively [32]. The band around 3000 cm^−1^ was attributed to the N-H stretching vibration of the APTES moieties [33]. For the PSAMs shown in Figure 3a, the band at 960 cm^−1^ was assigned to the Si-O in-plane stretching vibration of the Si-OH species [32]. The Si-OH species gradually disappeared from PSAMs, ASAMs and RSAMs. That revealed that the Si-OH groups in PSAMs were consumed by grafting APTES molecules in the following step, as illustrated in Figure 1b. For the ASAMs shown in Figure 3b, the obvious band around 1637 cm^−1^ could be the result of the bending vibration of the -NH_2_ groups in APTES [34], and the -NH_2_ groups gradually weakened in the RSAMs, indicating that they were successfully functionalized and crosslinked with styrene in the subsequent reaction. All of the RSAM samples obtained by the VD and SCD methods showed adsorption peaks at 1433, 719 and 699 cm^−1^ (the blue area in Figure 3), which derived from the deformation and twisted vibrations of Si-C_6_H_5_ [35,36]. With the increase in crosslinking time from 3 to 24 h, the adsorption peak of Si-C_6_H_5_ gradually increased and became sharper under both drying methods, indicating that more rigid phenyl groups were successfully introduced into the aerogel framework. Also, the adsorption peak at 1597 cm^−1^ was caused by the symmetric stretching vibration of C=C [37], which derived from the benzene ring, further illustrating the existence of the phenyl groups in the aerogel skeleton.

### 3.4. Thermal Performances

**Figure 4:**
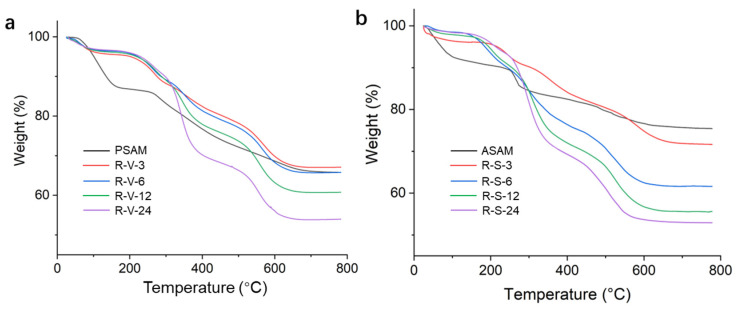
TGA curves of the SAMs obtained by different drying methods. (**a**) VD. (**b**) SCD.

Thermal stability is one of the most important properties for aerogels. Most tests are conducted in nitrogen conditions. However, for practical applications, recording the weight loss in air conditions makes more sense. TGA and DTA curves of the SAMs in air conditions are shown in Figure 4 and Figure S2. The magnitude of weight losses of various samples in different temperature ranges are listed in Table S1. RSAMs obtained via different drying methods showed very similar properties. For the RSAMs, there were four main stages that occurred on the curves. First, the weight loss below 200 °C derived from the adsorbed water. Both the PSAMs and ASAMs presented more weight loss of water than RSAMs, as they possessed abundant hydroxyl species. The hydroxyl groups could easily adsorb moisture via hydrogen bonding. There was no obvious weight loss before 200 °C for RSAMs, indicating that the phenyl species had successfully replaced the hydrophilic hydroxyl and amino groups. Second, the weight loss between 200 and 400 °C was ascribed to the pyrolysis of propyl and amino groups from APTES. Thus, the PSAMs and ASAMs showed the maximum weight loss at this stage. Third, the weight loss between 400 and 600 °C was mainly attributed to the decomposition of the polystyrene. Last, the curves after 600 °C gradually became stable, and the weight loss during this stage was caused by the organic moieties like alkyl groups, but was not so important and could be ignored. The DTA curves of various SAMs also confirmed that the phenyl groups in the RSAMs started to decompose from 400 to 600 °C. It was noted that the residual weight percentage of the RSAMs decreased with the increase in crosslinking time in the TGA curves, while the maximum degradation temperature did not change significantly in the DTA curves. However, considerable weight loss of the RSAMs further indicated the effective crosslinking of the phenyl groups onto the aerogel skeleton.

### 3.5. Microstructure and Morphology

**Figure 5:**
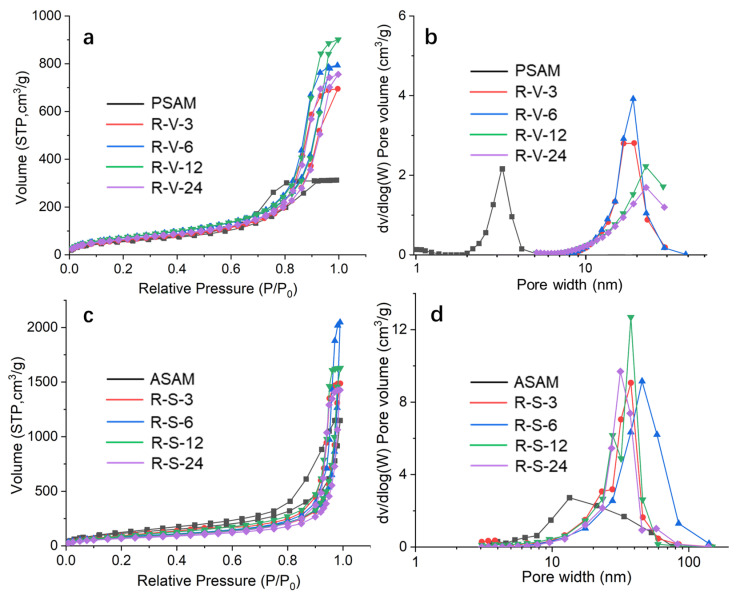
N_2_ adsorption/desorption isotherms and pore size distribution curves of RSAMs obtained by different drying methods. (**a**,**b**) VD. (**c**,**d**) SCD.

According to the definition by the International Union of Pure and Applied Chemistry (IUPAC), porous materials can be divided into three categories according to their pore sizes: microporous (<2 nm), mesoporous (2–50 nm) and macroporous (>50 nm). The pore structure information for the obtained aerogels was characterized using the specific surface area analyzer and the BJH method. The N_2_ adsorption/desorption isotherms and pore size distribution curves are shown in Figure 5. The corresponding physical properties of the aerogel, including the specific surface area and pore volume, are summarized in Table 1. It can be seen from Figure 5a,c that all of the aerogel samples showed type IV isotherms with H3 hysteresis loops, indicating that these aerogel samples had mesoporous structures composed of irregular spherical pores and nanoparticles, which was further confirmed by the pore size distribution curves in Figure 5b,d. More interestingly, a distinct peak that only occurred at the pore size of ca. 3 nm was observed for the PSAM in Figure 5b but not observed for other samples, implying that the pore size distribution of the material was uniform, and most of the pores were mesoporous with narrow pore size distributions, which is consistent with the pore structure characteristics of typical silica aerogels. Meanwhile, the crosslinking samples did not show those pore size distributions under 5 nm, indicating that polymer crosslinking induced irregular wide pore size distributions and potential large silt or even gaps between the generated polymers. There was no saturation adsorption platform close to 1 of P/P_0_ on the SCD method curves, indicating the existence of macropores and large voids in the RSAMs synthesized via the SCD method. On the contrary, the saturation adsorption platform occurred on the samples obtained via the VD method, demonstrating high mesoporosity of the samples synthesized via the VD method. These phenomena are consistent with the pore size distribution curves. Different drying methods have a great influence on the pore structure. The R-V-x samples in Figure 5b show typical mesopores ranging from 3 to 30 nm, while the pores of R-S-x samples are broader, in the range of 10 to 100 nm. This can be explained by the fact that the volume shrinkage in a vacuum atmosphere is more than that in a supercritical atmosphere [38], thus leading to a narrower and denser pore size distribution. Consequently, the apparent densities of the R-V-x samples were much higher than those of the R-S-x samples. Interestingly, it was found that the pore size and pore volume of the RSAMs were distinctly larger than those of the PSAMs, which seems incompatible with common sense. Considering that the PSAMs possessed the lowest apparent density of 0.08 g/cm^3^, their total pore volume was 27.5 cm^3^/g in theory, assuming a skeleton density of SiO_2_ is 2.2 g/cm^3^. The measured pore volume (0.49 cm^3^/g) was much lower than the theoretical pore volume, which means there were many pores that could not be detected by the N_2_ adsorption method. Generally, the N_2_ adsorption method only detects pores especially from 1 to 100 nm. The large voids that existed in PSAMs were partially filled after polymer crosslinking with polystyrene, thus most pores of RSAMs were nanoscale, which can be precisely detected by the N_2_ adsorption method. Moreover, besides the drying method, crosslinking time also influenced the specific surface area and pore volume significantly. According to Table 1, with the increase in crosslinking time from 3 to 24 h, the specific surface area of the RSAMs decreased gradually, changing from 261.1 to 218.2 m^2^/g for the VD method, and from 289.8 to 277.9 m^2^/g for the SCD method, and also showing the same decaying trend in the pore volumes. This could be due to the fact that some mesopores were filled and blocked during the polymer crosslinking process, which made a significant contribution to the calculation of specific surface area. In addition, the decrease in the porosity well confirmed the above conclusion. Nonetheless, the specific surface area and pore volume of RSAMs were higher than those of their state-of-the-art counterparts [39,40,41].

**Figure 6:**
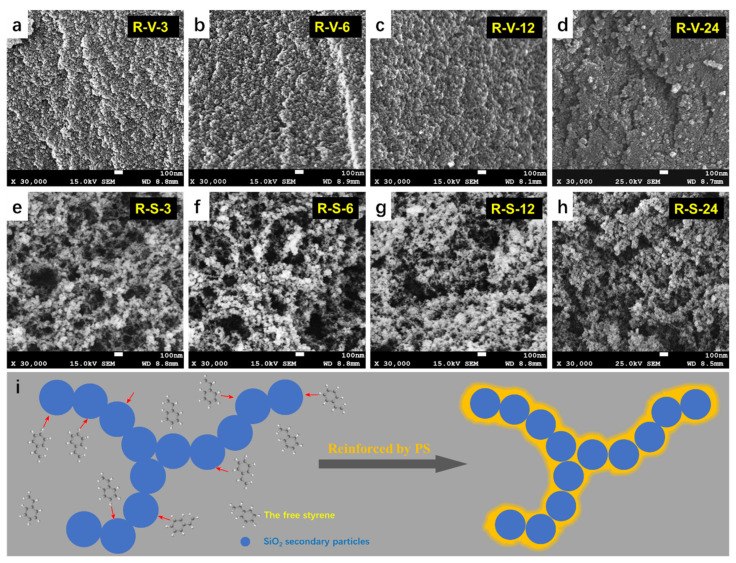
Microstructure of RSAMs obtained through different drying method under varying crosslinking times. (**a**–**d**) SEM images under vacuum drying for (**a**) 3 h, (**b**) 6 h, (**c**) 12 h, and (**d**) 24 h crosslinking. (**e**,**f**) SEM images under supercritical CO_2_ drying for (**e**) 3 h, (**f**) 6 h, (**g**) 12 h, and (**h**) 24 h crosslinking. (**i**) Schematic diagram of the formation of the polystyrene-reinforced silica aerogel skeleton.

The influence of the drying method and crosslinking time on the microstructure of the obtained aerogels was investigated. The results are shown in Figure 6a–h. All of the aerogel samples had nano-porous networks consisting of colloidal particles and mesopores. As expected, the microstructure was markedly influenced by the drying method. RSAMs obtained via vacuum drying had dense and compact networks in the absence of large voids, considering the large volume shrinkage during the drying process. However, RSAMs synthesized via supercritical CO_2_ drying had highly porous networks with apparent mesopores and large voids, which was similar to that reported for polymer crosslinked silica aerogels [42,43,44]. Meanwhile, the crosslinking time had a significant influence on the microstructure. With the increase in crosslinking time, the crosslinked polymer structure was more widely distributed and gradually wrapped the surface of the silica aerogel skeleton. Especially, when the crosslinking time was longer than 12 h, the aerogel exhibited a non-particulate structure, indicating that the introduction of polystyrene contributed to the transformation of the spherical particle structure to the non-particulate structure, which made the connection of the aerogel skeleton stronger. As vividly clarified in Figure 6i, condensation of free styrene can take place on the surface of the silica secondary particles. With the increase in crosslinking time, the reinforcement effect of phenyl groups from styrene on the aerogel skeleton was clearly increasing. As a result, the aerogel skeleton was transformed from a fragile particulate structure into an enhanced non-particulate structure.

### 3.6. Mechanical Performance

**Figure 7:**
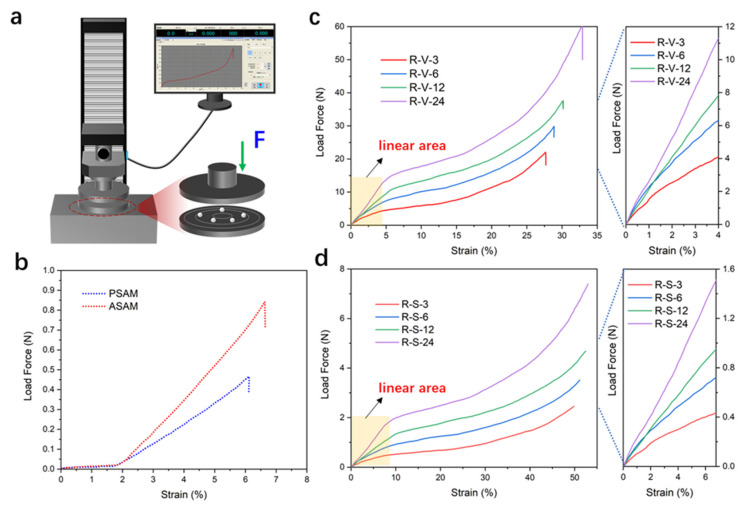
Mechanical performance of RSAMs. (**a**) Test device for spherical materials using 5-point method. (**b**) Compressive curves of PSAMs and ASAMs. (**c**) Compressive curves via VD method and the enlarged linear area. (**d**) Compressive curves via SCD method and the enlarged linear area.

In order to investigate the mechanical properties of the polymer-reinforced silica aerogels, a uniaxial compression test machine was used to test the force–strain (F-ε) curves, and the results are shown in Figure 7. Distinguished from regular cubes, cylinders and other regular monolithic structures, spherical materials present slight differences and also difficulty under the traditional stress–strain test method, considering the dynamically changing contact area of the spherical materials [45]. Here, five microspheres with average diameter of 3 ± 0.2 mm were selected to conduct the compression test. The test model is vividly shown in Figure 7a. To have a better understanding of the effect of the polymer crosslinking, the curves of un-crosslinked samples are presented in Figure 7b. The crack force of PSAM and ASAM was 0.45 and 0.85 N, respectively. Correspondingly, the allowed maximum deformation strain was 6.0 and 6.6%; the results are consistent with traditional brittle SiO_2_ aerogels [46]. The compressive curves of crosslinked samples via the vacuum drying method are shown in Figure 7c. Three characteristic stages can be recognized from the loading–unloading curves. The first stage, for ε < 5%, was a linearly elastic region under the low-pressure strain. The second stage, for 5% < ε < 25%, was a relatively flat stress plateau with slight hardening. The third stage, for ε > 25%, was a plastic densification and rapid decrease to the coming crack point. The sample crosslinked for 24 h possessed the maximum crack force of 60 N and also a high deformation strain of 32%. The slope (*k*) of the linear region can reflect the Young’s modulus of the materials to a certain degree, as seen in the enlarged picture in Figure 7c. For R-V-x samples, the *k* value gradually increased from 100 to 275 as the crosslinking time increased from 3 to 24 h, indicating that a more robust network was gradually forming in the aerogel skeleton. The compressive curves of the crosslinked samples via the supercritical CO_2_ drying method are shown in Figure 7d. The sample crosslinked for 24 h possessed a crack force of 7.2 N and maximum deformation strain of 50%. For the R-V-x samples, the *k* value gradually increased from 6 to 26 as the crosslinking time increased from 3 to 24 h. Compared to the vacuum drying samples, the R-S-x samples showed lower compressive force but exhibited higher deformation strain of up to 50% in general. This is understandable when the microstructure of the R-V-x and R-S-x samples are taken into consideration. The CO_2_ supercritical drying fortunately preserved the rich porous structure in the aerogel, and when the polymer crosslinking structure wrapped the surface of the secondary particles, it also enhanced the connection of the aerogel skeleton. In addition, polymer-reinforced aerogels not only exhibited robustness in compression resilience but also afforded a large deformation.

### 3.7. Adsorption and Recyclability

**Figure 8:**
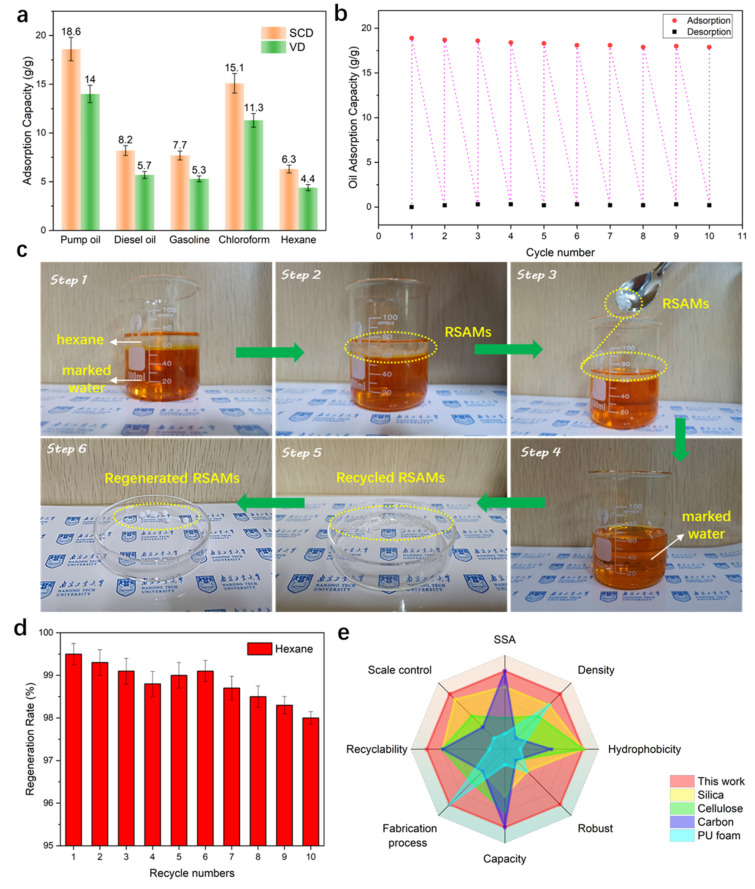
Adsorption and recyclable performance of RSAMs. (**a**) Adsorption capacity for several oils and organic solvents. (**b**) Repeated adsorption and desorption capacity of RSAMs for pump oil. (**c**) Organics removal from water: the recycling and regeneration process using RSAMs. Step 1 is the oil–water mixture system, step 2 is the adsorption process, step 3 is the removal process, step 4 is the system after removal, step 5 is the recycled RSAMs, step 6 is the regenerated RSAMs. (**d**) Regeneration rate of RSAMs for hexane. (**e**) Comprehensive performance comparison of RSAMs with various adsorbents for oil removal.

Hydrophobic silica aerogels are commonly used for oil removal from water and have already shown good potential. The oil adsorption capacities of hydrophobic RSAMs selected from R-S-24 and R-V-24 were measured. Figure 8a shows the adsorption capacity of hydrophobic RSAMs for several oils and organic solvents, including pump oil, diesel oil, gasoline, chloroform and hexane. As seen in Figure 8a, R-S-24 exhibits outstanding sorption capacity for both oils and organic pollutants, ranging from 6.3 to 18.6 g/g, which is higher than that of R-V-24 in general. RSAMs obtained via supercritical CO_2_ drying possessed better oil adsorption capacities than those obtained via vacuum drying. The reason is that the RSAMs synthesized via supercritical CO_2_ drying have more pore volume, porosity, and macropores, which can provide more spaces for oil holding and enhance the mass transfer in the network. The phenomena are more obvious for oils than for hexane, which is attributed to the fact that the oils have much higher viscosity than hexane, which restricts the mass transfer more significantly for aerogel samples with smaller pores. The optimal oil adsorption capacity of RSAMs is 18.6 g/g, which is higher than that of isocyanate-based silica aerogel [47], cellulose aerogel [48], carbon aerogel [49], PU foam [50] and other state-of-the-art counterparts, as listed in Table 2. Figure 8b shows the repeated adsorption and desorption capacity of R-S-24 samples for pump oil; the absorption capacity of the aerogel declined slightly after repeating the process for 10 cycles, suggesting the as-prepared aerogel still exhibited a stable absorption capacity. Figure 8c shows the separation, recycling and regeneration process of R-S-24 samples for a mixture of hexane and water stained with methyl orange. As shown in step 1, the transparent oil was on the top, while the water marked by methyl orange was on the bottom. When R-S-24 samples were added in the oil–water mixture in step 2, they only floated on the oil layer rather than sinking into the water layer, due to their exceptional hydrophobicity and low density. Therefore, they could be easily collected through either dredging or filtration in step 3. And after several cycles of adsorption, the hexane in the water was completely removed, as shown in step 4. After adsorption, these aerogels became a little bit transparent, the adsorbed liquids were well stored in the abundant nanopores of the aerogels, and no dripping liquids were observed during the whole process in step 5. Then, the recycled aerogels from the former step were first washed by ethanol and dried by a rapid vacuum drying method to remove the inner liquids efficiently. The regenerated samples still retained the original appearance in step 6, which was attributed to the outstanding compressive strength and robustness of the polymer-reinforced skeleton. Regenerability is significant for a sorbent, which can reduce its long-running cost. As shown in Figure 8d, the cyclic hexane absorption capacities of the R-S-24 samples within 10 cycles did not show obvious depletion, and could still maintain a high value of 98%, indicating that the RSAMs are both structure- and performance-stable sorbents. Figure 8e and Table 2 provides a comprehensive comparison of RSAMs with various adsorbents for oil removal. Compared with other adsorbent materials like traditional silica aerogel [47], cellulose aerogel [48], carbon aerogel [49] and PU foams [50] both from reported literatures and commercial fields, as summarized in Table 2, the optimal RSAMs have a more comprehensive excellent performance in terms of both structure and practical performance such as specific surface aera (281 m^2^/g), density (0.16 g/cm^3^), hydrophobicity (126°), robust mechanical performance, abundant oil capacity (18.6 g/g), fabrication process (both VD and SCD), size controllable (show as microspheres) and stable recyclability, which is expected to be a promising renewable absorption material with potential applications in oil pollution treatment.

## 4. Conclusions

In summary, robust hydrophobic polymer-reinforced silica aerogel microspheres (RSAMs) were successfully fabricated by a facile approach. Both vacuum drying and supercritical CO_2_ drying were used to synthesize the RSAMs. However, the volume shrinkage of the RSAMs obtained via the vacuum drying method was much greater than that via the supercritical CO_2_ drying method, leading to higher apparent densities. The polymer crosslinking time also significantly affected the microstructure and the pore structure. Longer crosslinking times led to more volume shrinkage, thus resulting in a larger apparent density, lower pore volume, narrower pore size distribution and more compact network. Notably, the hydrophobicity increased with the increase in crosslinking time. After polymerization for 24 h, the RSAMs possessed a highest water contact angle of 126°. Owing to their excellent hydrophobicity, the RSAMs via supercritical CO_2_ drying exhibited a maximum oil adsorption capability of 18.6 g/g, higher than that of their state-of-art counterparts. More importantly, polymer crosslinking enhanced the robustness of the skeleton of brittle aerogels effectively. Moreover, their robust mechanical properties ensured excellent reusability and recyclability, allowing for multiple oil adsorption–desorption cycles without significant degradation in performance. The novel sorbent preparation method is facile and inspiring, and the resulting robust and hydrophobic RSAMs are exceptional in capacity, efficiency, stability and regenerability.

## Figures and Tables

**Table 1:** The physical properties of the obtained aerogels.

Sample	Apparent Density (g/cm^3^)	Porosity (%)	Specific Surface Area (m^2^/g)	Pore Volume (cm^3^/g)	Drying Method	Hydrophobic Properties
PSAM	0.080	96.4	203.6	0.49	VD ^a^	no
ASAM	0.137	93.8	230.4	2.78	SCD ^b^	no
R-V-3	0.158	92.8	261.1	1.39	VD	no
R-V-6	0.166	92.5	256.1	1.23	VD	no
R-V-12	0.191	91.3	240.6	1.16	VD	no
R-V-24	0.210	90.5	218.2	1.07	VD	yes
R-S-3	0.141	93.6	289.8	3.81	SCD	no
R-S-6	0.148	93.3	285.7	3.77	SCD	no
R-S-12	0.155	93.0	280.5	3.68	SCD	no
R-S-24	0.163	92.6	277.9	3.52	SCD	yes

^a^ Vacuum drying. ^b^ Supercritical CO_2_ drying.

**Table 2:** Comparison of various adsorbents for oil removal.

Sample	Adsorption Capacity (g/g)	Specific Surface Area (m^2^/g)	Apparent Density (g/cm^3^)	Hydrophobicity (°)	Fabrication Process	Robust Level	Ref.
Silica aerogel	6.0	296	0.08	128	SCD	medium	Sun et al. [47]
Cellulose aerogel	12.0	154	0.05	140	SCD	low	Zhao et al. [48]
Carbon aerogel	17.1	309	0.03	99	FD	low	Li et al. [49]
PU foam	7.3	not reported	0.06	95	VD	low	Dat et al. [50]
RSAMs	18.6	281	0.16	126	VD/SCD	high	This work

## Data Availability

Not applicable.

## Supplementary Materials

The following supporting information can be downloaded at: https://www.mdpi.com/article/10.3390/polym15173526/s1, Figure S1: Samples obtained at longer polymerization time of 36 and 48 h; Figure S2: DTA curves of the SAMs under different drying method. (a) VD. (b) SCD; Figure S3: High temperature oil adsorption ability of RSAMs; Figure S4: FTIR spectra of the RSAMs under high temperature; Table S1: Weight losses in different temperature ranges.
