# Supplementary material for: Facile Synthesis of Polymer-Reinforced Silica Aerogel Microspheres as Robust, Hydrophobic and Recyclable Sorbents for Oil Removal from Water
Source: Polymers (Basel). 2023 Aug 24;15(17):3526. doi: 10.3390/polym15173526 (PMC10489638; doi:10.3390/polym15173526)
Supplement: Supplementary file 1 [file polymers-15-03526-s001.zip › polymers-2561177-supplementary.pdf]

Supplementary Materials

Facile Synthesis of Polymer-Reinforced Silica Aerogel Microspheres as Robust, Hydrophobic and Recyclable Sorbents for Oil Removal from Water

Zhiyang Zhao [1,2,3,4,†], Jian Ren [1,2,3,†], Wei Liu [1,2], Wenqian Yan [1,2], Kunmeng Zhu [1,2], Yong Kong [1,2,3,*], Xing Jiang [1,2] and Xiaodong Shen [1,2,3,*]

1 College of Materials Science and Engineering, Nanjing Tech University, Nanjing 210009, China
2 Jiangsu Collaborative Innovation Center for Advanced Inorganic Function Composites, Nanjing 210009, China
3 State Key Laboratory of Materials-Oriented Chemical Engineering, Nanjing 210009, China
4 Swiss Federal Laboratories for Materials Science and Technology, EMPA, Überlandstrasse 129, 8600 Dübendorf, Switzerland
* Correspondence: ykong@njtech.edu.cn (Y.K.); xdshen@njtech.edu.cn (X.S.)
† These authors contributed equally to this work.

1. Supplementary Figures

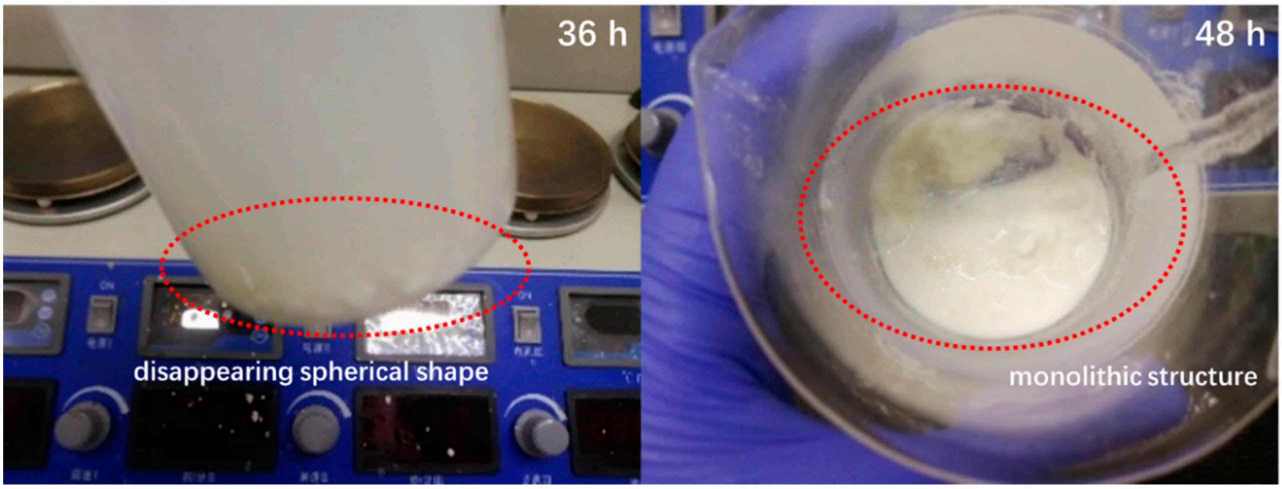

Figure S1. Samples obtained at longer polymerization time of 36 and 48 hours.

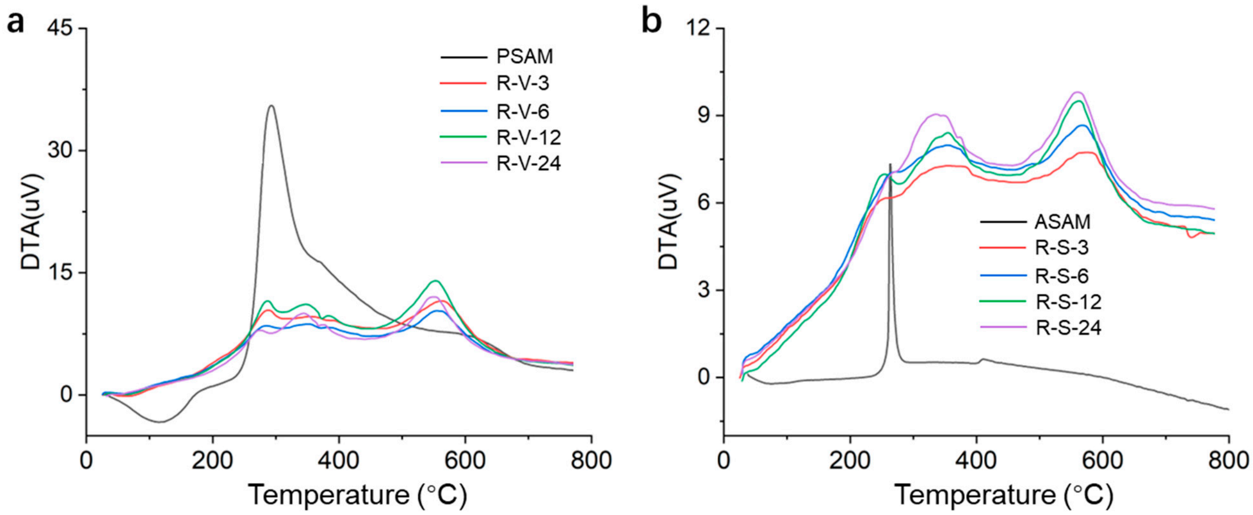

Figure S2. DTA curves of the SAMs under different drying method. (**a**) VD. (**b**) SCD.

2. High Temperature Oil Adsorption Test and Chemical Stability

Temperature resistance is one of the fascinating properties of aerogels distinguished from other materials. So, we added the oil adsorption experiment using our samples at a high temperature of 120°C, as shown in the pictures below, the silica aerogel can both maintain

good shape, hydrophobicity, and adsorb oil from oil-water mixture system efficiently, showing a good high temperature adsorption ability. We select the most two hydrophobic aerogel samples of R-V-24 and R-S-24 to do the thermal treatment at a high temperature of 200°C in the muffle furnace, in air atmosphere, hold two hours at the maximum temperature. The samples after high temperature treatment are denoted as R-V-200 and R-S-200, while the untreated samples are denoted as R-V and R-S, respectively. Their chemical stability was investigated by Fourier transform infrared spectra (FTIR). The curves are similar after high temperature, the blue area is the characteristic peak of the benzene ring structure, they can still be observed after high temperature, indicating the chemical stability of silica aerogel at a high temperature.

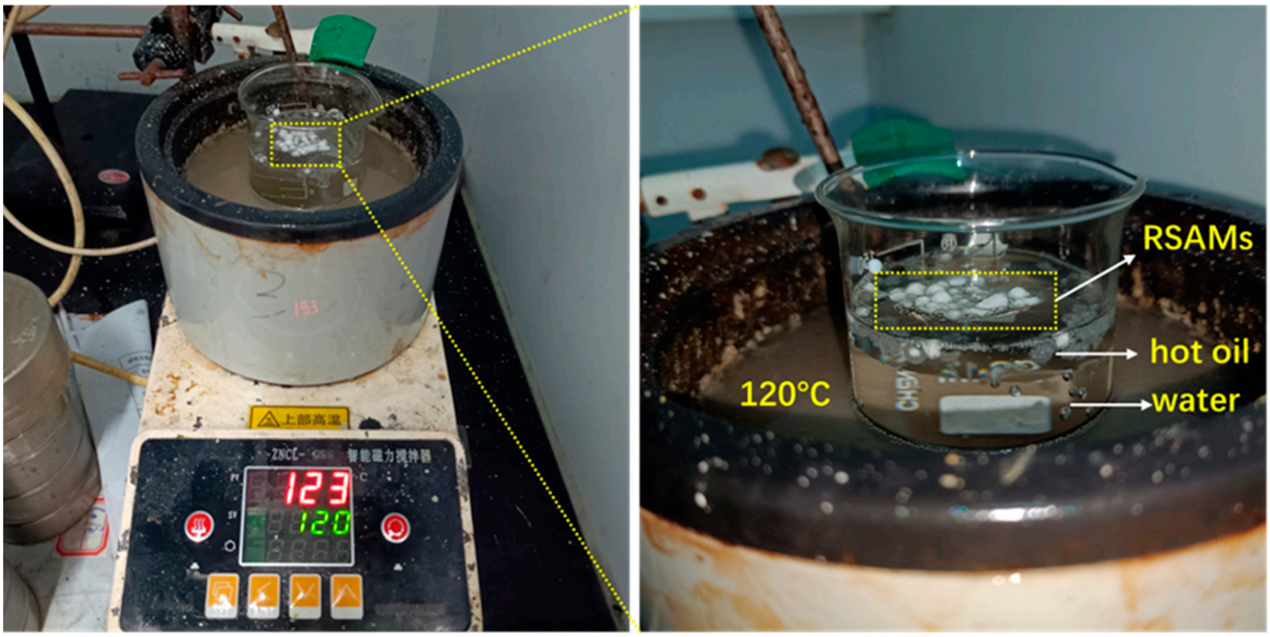

Figure S3. High temperature oil adsorption ability of RSAMs.

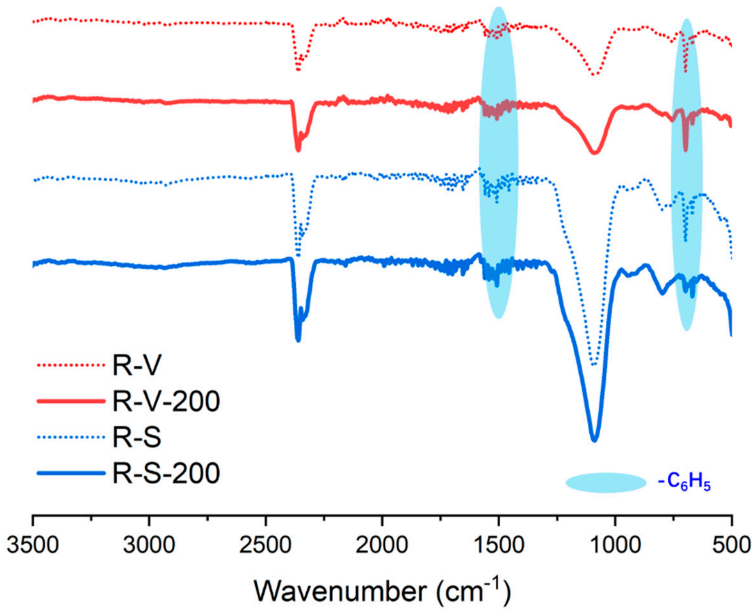

Figure S4. FTIR spectra of the RSAMs under high temperature.

3. Supplementary Table

Table S1. Weight losses in different temperature ranges.

	<200°C	200-400°C	400-600°C	>600°C	Remaining
PSAM	12%	10%	10%	2%	66%
R-V-3	4%	13%	13%	2%	68%
R-V-6	3%	16%	13%	2%	66%
R-V-12	3%	20%	14%	2%	61%
R-V-24	3%	27%	14%	2%	54%
ASAM	10%	8%	5%	2%	75%
R-S-3	3%	13%	10%	2%	72%
R-S-6	5%	19%	14%	1%	61%
R-S-12	5%	23%	15%	1%	56%
R-S-24	4%	26%	17%	1%	52%
